# Dr. Osler’s Little “Joke”

**Published:** 1905-04

**Authors:** 


### TEXAS MEDICAL JOURNAL.

AUSTIN, TEXAS.

A MONTHLY JOURNAL OF MEDICINE AND SURGERY.

EDITED AND PUBLISHED BY
F. E. DANIEL, M. D.
Mrs. F. E. DANIEL, Business Manager.

Published Monthly at Austin, Texas. Subscription price $1.00 a year in advance.

Eastern Representative: John Guy Monihan, St. Paul Building, 220 Broadway New York City.

Official organ of the West Texas Medical Association, the Houston District Medical Association, the Austin District Medical Society, the Brazos Valley Medical Association, the Galveston County Medical Society, and several others.

### DR. OSLER'S LITTLE "JOKE."

"I have saw *young* men that knowed it all."
—*James Whitcomb Riley.*

**He did not know it was loaded.**

I dare say that no one was more surprised than the celebrated professor himself — (now Regius Professor of Medicine at the University of Oxford)—at the storm that has been raised by his valedictory at Johns Hopkins. It has been a storm of mixed indignation, protest and ridicule. The Chicago Octopus—(the *Journal A. M. A.*)—says "he didn't say it." Prof. Edwin G. Dexter, of the University of Chicago (in *Popular Science Monthly* for April), says he was "joking," and that "it (the joke) proved too subtle for many a yellow sheet." It has proved "too subtle" for sheets of other colors, also (red, for instance), if we may judge by the seriousness with which most papers discuss it.

Perhaps Professor Dexter's construction is the most charitable; else, it were puerile. Such levity is out of place—if it be levity—and is as unbecoming, to, and was as unexpected from such a man, as it would be if it had been uttered as a statement of fact, for it is not true.

That men over 60 are useless and ought to get off the earth *via* the chloroform route, and that the work of the world is done by men under 40, is a statement easily refuted. In contradiction, the names at once suggest themselves of Virchow, Bismarck, and Von Moltke, Spencer, and Darwin, Rockefellow, and Morgan, Gladstone, the late Pope Leo, "Bobs" (General Roberts), and all the Japanese generals, and they are not exceptions, but the rule.

Dr. Osler's observations tally exactly with those of James Whitcomb Riley. He has "saw young men that knowed it all," but they are in the minority. Professor Dexter, notwithstanding he endeavors to screen the great professor by throwing the mantle of charity over his silly utterances, and calling it a "joke," nevertheless proceeds to treat it seriously, and to utterly refute it, so far as a tabulation of some 7000 names can refute it—names of persons who have gained distinction, and celebrity, and public recognition—a list embracing actors, artists, authors, business men, clergymen, college professors, congressmen, editors, engineers, financiers, inventors, lawyers, librarians, physicians, musicians, sailors, soldiers, scientists, and statesmen. In this list, the aggregate of which is 6983, the *average* age at which all classes of men become noted was 54, and the percentage under 40 (Dr. Osler's limit of usefulness), was 16. That is, 84 per cent were over 40. Of 200 business men (successes), the *average* age was 63. Of 215 financiers, the *average* age was 64. Of 26 inventors, the *average* age was 62. Of 205 soldiers, the *average* age was 63. Of 540 physicians, the *average* age was 56. To average thus, indicates that if even 16 per cent are under 40, a very large number must be up into the 80's and 90's.

But what's the use to argue it seriously? Dr. Osler knows that history does not bear him out in the assertion. If it were intended for a "joke," it reminds me of the last joke of a wag of an Irishman,—a friend of mine,—who plugged with powder the candles to be used at his wake, so that at about midnight "there was somethin' doin'." His "wakers," who were giving him a first-class send-off—as were Dr. Osler's friends—could not have been more astonished at the ghastly and untimely joke than have been the friends of the great Dr. Osler at his startling pyrotechnics, (Note the similarity of the two occasions), called by his apologists a "joke," and by the rest of the world an absurd and silly utterance. Can it be that the doctor is rare ripe, and is already showing signs of senility?

For the information of my readers, I give below the text of the now notorious utterance, with an apology for devoting so much space to it.

The portion of his address that has brought him so prominently into the public eye had to do with the age of greatest usefulness in man, and runs as follows:

"I have two fixed ideas, well known to my friends, harmless obsessions, with which I sometimes bore them, but which have a direct bearing on this important problem. The first is the comparative uselessness of men above forty years of age. This may seem shocking, and yet, read aright, the world's history bears out the statement. Take the sum of human achievement in action, in science, in art, in literature—subtract the work of the men above forty, and while we should miss great treasures, even priceless treasures, we should practically be where we are today. It is difficult to name a great and far-reaching conquest of the mind which has not been given to the world by a man on whose back the sun was still shining. The effective, moving, vitalizing work of the world is done between the ages of twenty-five and forty—these fifteen years of plenty, the anabolic or constructive period, in which there is always a balance in the mental bank, and the credit is still good. * * *

"My second fixed idea is the uselessness of men above sixty years old, and the incalculable benefit it would be in commercial, political and in professional life if, as a matter of course, men stopped work at this age. Donne tells us in his 'Biathanatos' that, by the laws of certain wise states, sexagenari were precipitated from a bridge, and in Rome men of that age were not admitted to the suffrage, and they were called *deponati,* because the way to the senate was *per pontem*, and they, from age, were not permitted to come hither. In that charming novel, 'The Fixed Period,' Anthony Trollope discusses the practical advantage in modern life of a return to this ancient usage, and the plot hinges upon the admirable scheme of a college, into which, at sixty, men retired for a year of contemplation before a peaceful departure by chloroform. That incalculable benefits might follow such a scheme is apparent to any one who, like myself, is nearing the limit, and who has made a careful study of the calamities which may befall men during the seventh and eighth decades."

Does that look like a "joke"? To a man up a tree, it looks like a "fixed idea." He says it is a fixed idea—been there a long time. Exit Osler.

---

THIS issue of the "Red Back" commemorates the thirty-seventh annual meeting of our big State Medical Association (Houston, April 24-28), with an enrolled membership of nearly 3000. It is expected there will be a large attendance, and that many delegates will be accompanied by the ladies of their families. The House of Delegates will meet at 2 p. m. Monday, April 24th. The general session will begin Tuesday, 25th, at 10 a. m. I do not publish the program, because it makes twenty-four pages, and I have not room for it, and it is unnecessary, in as much as 3500 copies of it in pamphlet form have been sent out by the secretary, and everyone of my subscribers who will see this number of the JOURNAL will have received the program in advance of this publication. There are 120 papers—twenty-two in the surgical section alone. It is most elaborate, and will prove most interesting. The social features will be very attractive, and Houston will be in her Easter attire and as gay as a jaybird. The meeting will be one of great importance to the society because of the remarkable temper of the Legislature with reference to protective legislation and the disposition to recognize every pathy down to Voodoo Sam.

---

I HAVE mailed every subscription bill on my books. It is a small matter of a dollar or so to each subscriber, but the aggregate is a large sum, and I ask those who will not be at Houston to *remit;* and those who will attend the meeting are requested, and will be expected, to walk up to the "Mascot's" desk and *ante.* At the sign of the "Red Back."

---

SENTIMENT prevails in the Texas Legislature against common sense. Sentiment killed the Anatomical bill. One fellow said: "No, I can never vote to condemn, by a law, a poor devil to the dissecting table because he had the misfortune to die in a poorhouse or charity hospital." That was Terrell, of Morris county. Another (Senator Davidson, of Cuero) jokingly, wanted to amend the bill so as to include the corpses of all doctors. The bill was smothered in the Senate. The facetious "grave and reverend signors" call it the "Ghost bill." That educated gentlemen should so ridicule such a bill is almost incredible. The great State of Texas founded a great medical college, pays a professor of anatomy $4000 a year to teach anatomy, requires its graduates to have a thorough knowledge of anatomy and to stand examination for the M. D. degree, and another for license to practice, yet *makes it a felony* to procure the material for the study and demonstration of anatomy, the basis of medical education. We have gone back to the dark ages in the matter of sentiment. This bill passed last session, but Sayers vetoed it.

---

DR. GEO. H. LEE, councilor for the Ninth District, resigned because of interests that called him to Mexico. The president has not appointed a successor, but leaves it to the House of Delegates to fill the vacancy.

---

NO MEDICAL LEGISLATION has been enacted up to this writing (April 5th). A bill regulating the practice of pharmacy passed, creating a board of examiners. It is thought bills now pending for separate boards for "pathies" will not get through, as adjournment is close at hand.

---

THE AMERICAN MEDICO-PSYCHOLOGICAL ASSOCIATION will meet at San Antonio, April 18, inst. This is an association of the asylum superintendents of the United States, which was organized in 1844. The name was changed in 1893. It is expected that there will be a large attendance, and the meeting is looked forward to with great interest, especially in view of the immense and rapid increase in insanity. I have not received the program. The president of the State Association will welcome the members on behalf of the profession of Texas. Many of the celebrities of America will be present.

---

APROPOS OF REGULATING THE PRACTICE, Horne Tooke says (*The Pantheon*): "Æsculapius was so proficient in the practice of medicine that he restored to health the sick, and gave safety to those whose condition was desperate. He was thought to have the power of recalling the dead to life again. Virgil tells us that, "upon this, Pluto, the King of Hell, complained to Jupiter that his revenue was very much diminished. By his persuasion Jupiter killed him with a stroke of thunder." We have here, doubtless, the first recorded instance of an effort to regulate the practice of medicine. Let the Texas Solons make a note of it. P. S.: It has been learned later, upon reliable authority, that Dr. Æsculapius used Pe-ru-na exclusively in his practice, by means of which he cured even mild cases of death. Hence Pluto's kick.

